# Antioxidant and Anti-Glycation Potential of H2 Receptor Antagonists—In Vitro Studies and a Systematic Literature Review

**DOI:** 10.3390/ph16091273

**Published:** 2023-09-08

**Authors:** Grzegorz Biedrzycki, Blanka Wolszczak-Biedrzycka, Justyna Dorf, Daniel Michalak, Małgorzata Żendzian-Piotrowska, Anna Zalewska, Mateusz Maciejczyk

**Affiliations:** 1 Hospital Pharmacy, Provincial Specialist Hospital in Olsztyn, 10-900 Olsztyn, Poland; 2 Department of Psychology and Sociology of Health and Public Health, University of Warmia and Mazury in Olsztyn, 10-900 Olsztyn, Poland; 3 Department of Clinical Laboratory Diagnostics, Medical University of Bialystok, 15-089 Bialystok, Poland; 4 Students Scientific Club “Biochemistry of Civilization Diseases”, Department of Hygiene, Epidemiology and Ergonomics, Medical University of Bialystok, 15-089 Bialystok, Poland; 5 Department of Hygiene, Epidemiology and Ergonomics, Medical University of Bialystok, 15-089 Bialystok, Poland; 6 Experimental Dentistry Laboratory, Medical University of Bialystok, 15-089 Bialystok, Poland

**Keywords:** H2 receptor antagonists, H2-antihistamines drugs, antioxidant, anti-glycation, AGE, oxidative stress, glycoxidation

## Abstract

**Background:** Histamine H2 receptor antagonists are a group of drugs that inhibit gastric juice secretion in gastrointestinal diseases. However, there is evidence to suggest that H2 blockers have a broader spectrum of activity. The antioxidant properties of H2 blockers have not been fully elucidated, and their anti-glycation potential has not been studied to date. Therefore, this is the first study to compare the antioxidant and antiglycation potentials of the most popular H2 antagonists (ranitidine, cimetidine, and famotidine) on protein glycoxidation in vitro. **Methods:** Bovine serum albumin (BSA) was glycated using sugars (glucose, fructose, galactose, and ribose) as well as aldehydes (glyoxal and methylglyoxal). **Results:** In the analyzed group of drugs, ranitidine was the only H2 blocker that significantly inhibited BSA glycation in all tested models. The contents of protein carbonyls, protein glycoxidation products (↓dityrosine, ↓N-formylkynurenine), and early (↓Amadori products) and late-stage (↓AGEs) protein glycation products decreased in samples of glycated BSA with the addition of ranitidine relative to BSA with the addition of the glycating agents. The anti-glycation potential of ranitidine was comparable to those of aminoguanidine and Trolox. In the molecular docking analysis, ranitidine was characterized by the lowest binding energy for BSA sites and could compete with protein amino groups for the addition of carbonyl groups. H2 blockers also scavenge free radicals. The strongest antioxidant properties are found in ranitidine, which additionally has the ability to bind transition metal ions. The systematic literature review also revealed that the anti-glycation effects of ranitidine could be attributed to its antioxidant properties. **Conclusions:** Ranitidine showed anti-glycation and antioxidant properties. Further research is needed, particularly in patients with diseases that promote protein glycation.

## 1. Introduction

Oxidative stress is defined as an imbalance between the generation of reactive oxygen species (ROS) and antioxidants in the body [1]. This process is inextricably linked to protein glycation, during which covalent bonds are formed between aldehyde groups in sugars and amino groups in proteins (known as the Maillard reaction or the browning reaction). The produced Schiff bases are highly unstable, and they are rapidly converted to Amadori products [2]. Amadori products undergo polymerization, polycondensation, and cross-linking reactions to form advanced glycation end-products (AGEs). These compounds are characterized by high biological reactivity. AGEs bind to specific receptors (RAGE) on cell surfaces [3], which activates the nuclear factor-kappa B (NF-κB) signaling pathway. NF-κB overexpression leads to the excessive production of not only cytokines, chemokines, and growth factors, but also ROS [4]. Protein glycation and oxidation are closely linked processes that are jointly referred to as glycoxidation. Interestingly, the underlying mechanisms of advanced glycoxidation are not only endogenous. Dicarbonyl derivatives (glyoxal (GO) and methylglyoxal (MGO)) and AGEs are also ingested with highly processed foods [5], which is why protein glycation has attracted considerable interest in research on lifestyle diseases. Numerous studies have shown that ROS and AGEs modify cell structures and functions, which can even lead to DNA damage and apoptosis [6,7,8]. Oxidative and carbonyl stress are implicated in the pathogenesis of peptic ulcer disease and gastritis [9]. Intensified peroxidation of gastric mucosa lipids has been observed in patients with gastritis and duodenitis. This process leads to the release of intracellular granules, such as lysosomal enzymes, resulting in further damage [4]. Glycation products promote epithelial damage in the gastrointestinal tract by degrading the basement membrane and inducing DNA damage [10]. Higher concentrations of antioxidants/anti-glycation agents in the stomach or a slower rate of ROS production can counteract these negative effects [11].

**Figure 1:**
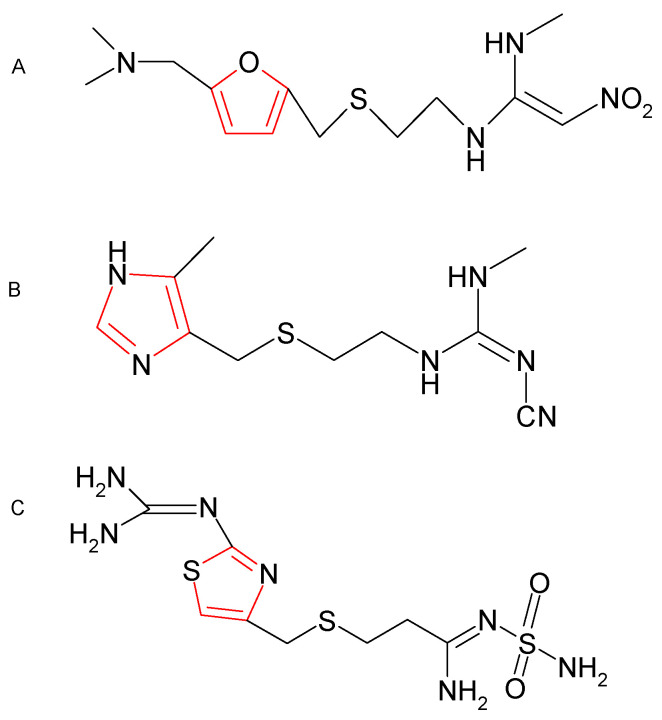
Chemical structure of the analyzed H2 receptor antagonists: ranitidine (**A**), cimetidine (**B**), and famotidine (**C**).

Histamine H2 receptor antagonists, mostly cimetidine (1-Cyano-2-methyl-3-(2-{[(4-methyl-1H-imidazol-5-yl)methyl]sulfanyl}ethyl)guanidine), ranitidine ((E)-N-{2-[({5-[(Dimethylamino)methyl]-2-furyl}methyl)sulfanyl]ethyl}-N’-methyl-2-nitro-1,1-ethenediamine), and famotidine (3-[[[[2-((Diaminomethylene)amino]thiazol-4-yl]methyl]thio]-N’-sulfamoylpropanimidamide), are used in the treatment of gastrointestinal inflammations (Figure 1) [12]. H2 blockers differ in chemical structure, but have a similar mechanism of action [13,14]. Cimetidine, ranitidine, and famotidine act as competitive antagonists that reversibly block H2 receptors on the basolateral membrane of gastric epithelial cells. Cimetidine and histamine are imidazole derivatives. Ranitidine and famotidine have different five-membered heterocyclic rings: ranitidine has a furan ring, whereas famotidine has a thiazole ring [15]. Both compounds have similar mechanisms of action, but their antagonistic potency is influenced by their structure. Ranitidine is 3–11 times more potent, and famotidine is 20–27 times more potent than cimetidine [14]. H2 blockers are recommended in the treatment of upper digestive tract disorders caused by an excessive decrease in the pH of the gastric acid [16]. H2 antagonists are also administered to patients undergoing cardiovascular surgery, neurosurgery, and organ transplantation to prevent stress ulcers [17]. It has been suggested that H2 antagonists could also have antioxidant potential. Such effects have been observed in patients with gastric ulcers treated with H2 blockers [18,19]. Other studies demonstrated that H2 blockers are potent scavengers of ROS produced by inflammatory cells, such as neutrophils [18,19]. On the other hand, cimetidine administered to patients with peptic ulcers and healthy subjects increased ^•^O_2_^−^ production in granulocytes, whereas ranitidine and famotidine did not elicit such a response [20]. In view of the contradictory reports on the antioxidant properties of H2 antagonists and a general scarcity of research on their effects on carbonyl stress, the present study was undertaken to investigate the anti-glycoxidant properties of these pharmaceutical products. In addition, it remains unknown whether the postulated anti-glycoxidant activity of H2 blockers should be attributed to their effect on intracellular redox homeostasis/inflammatory responses or their chemical structure. The answer to this question could have significant implications for clinical practice. This is the first study to compare the antioxidant and anti-glycation potentials of the most popular H2 antagonists—ranitidine, cimetidine, and famotidine.

## 2. Results

### 2.1. Systematic Review 

The literature review was conducted according to PRISMA 2020 criteria. A description of the inclusion and exclusion criteria is provided in the Materials and Methods. 

Three independent researchers performed an initial data analysis based on manuscript titles and abstracts. In the next stage, the selected articles were read and assessed for eligibility based on the adopted inclusion/exclusion criteria. Cohen’s kappa coefficient (κ) (κ = 0.91) was used to measure inter-rated reliability. To ensure high data quality, all manuscripts were assessed for methodology, and the following data were gathered: author names and affiliations, year of publication, research project, sample size, inclusion and exclusion criteria, study duration, and results. 

The systematic review yielded 868 research articles in the Medline database (PubMed). Of those, 782 were rejected due to misleading titles. Eighty-six abstracts were read, and twenty-two of those were assessed for eligibility based on the adopted inclusion/exclusion criteria. Fourteen articles were unrelated to the studied topic. Ultimately, only eight research articles were included in the study. A flowchart of the systematic review process is presented in Figure S1.

The results of the literature review indicate that cimetidine and famotidine inhibit the production of superoxide (^•^O_2_^−^) and hydrogen peroxide (H_2_O_2_) in human neutrophils [21]. Ranitidine exerts indirect antioxidant effects by inhibiting neutrophil activation (decreased production of neutrophil elastase and ^•^O_2_^−^) [22], as well as reducing tumor necrosis factor α (TNF-α) production by lipopolysaccharide (LPS)-stimulated monocytes [22]. Ranitidine, cimetidine, and famotidine also scavenge hydroxyl radicals (^•^OH) in various in vitro models [23,24]. The antioxidant properties of H2 blockers have also been confirmed in in vivo studies. Ranitidine scavenged 1,1-diphenyl-2-picrylhydrazyl (DPPH) radicals more effectively than famotidine and cimetidine [19]. H2 antagonists also effectively scavenged nitric oxide (^•^NO) radicals. Famotidine is a more potent scavenger of ^•^NO radicals than ranitidine and cimetidine [19]. Ranitidine exhibits antioxidant activity by inhibiting TNF-α production in the hepatocytes of rats with ischemia/reperfusion [25], as well as decreases lipid peroxidation in gastric mucosal injury induced by water immersion-restraint stress [22]. 

The detailed results of the literature review are presented in the Supplementary Material (Table S1).

### 2.2. The Effect of H2 Inhibitors on Protein Glycoxidation, Glycation, and Oxidative Damage in Glucose (Glc)-Induced Albumin Glycation

Various glycation agents, including sugars (glucose (Glc), fructose (Fru), galactose (Ga), and ribose (Rib)) and aldehydes (glyoxal (GO) and methylglyoxal (MGO)) were used in this study due to differences in the glycation kinetics of BSA. Human albumin is glycated mainly by D-glucose [26]. In the human body, glycation is a very long process (that lasts several weeks or even months) because only small amounts of Glc with a free aldehyde group are present in the blood plasma and tissues [27,28]. Gal, Rib, dicarbonyl compounds (GO), and their methylated derivatives (MGO) are significantly more reactive than Glc [9]. In the present study, glycation agents were applied at much higher concentrations than the physiological levels, but their doses were determined in biokinetic studies (proportionally to the concentrations of the tested substances) to simulate physiological processes in a shorter period of time [29,30,31,32]. The rate of glycation inhibition by H2 blockers was assessed by measuring the contents of glycoxidation (dityrosine and N-formylkynurenine), glycation (Amadori products and AGEs), and oxidation protein products (protein carbonyls (PCs) and total thiols (TTs)). Two reference substances were used to compare the results for ranitidine, cimetidine, and famotidine: aminoguanidine (a popular inhibitor of protein glycation) and Trolox (inhibitor of protein oxidation).

**Figure 2:**
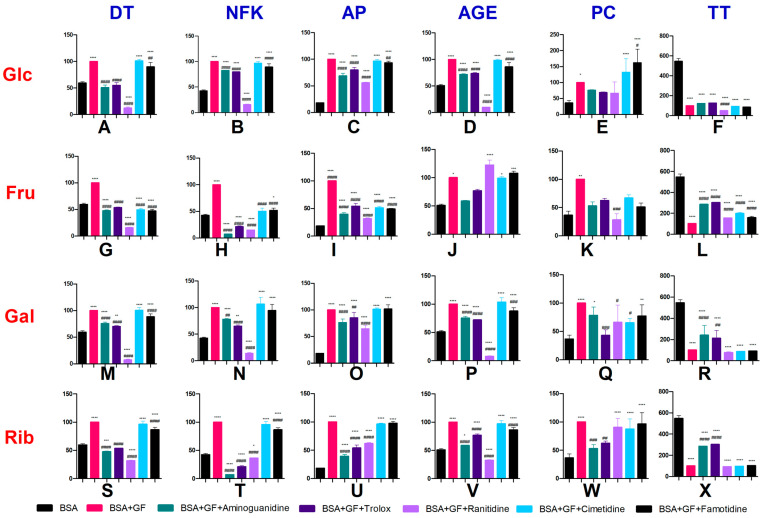
The effect of H2 inhibitors on protein glycoxidation (**A**,**B**,**G**,**H**,**M**,**N**,**S**,**T**), glycation (**C**,**D**,**I**,**J**,**O**,**P**,**U**,**V**), and oxidative damage (**E**,**F**,**K**,**L**,**Q**,**R**,**W**,**X**) in sugar-induced albumin glycation. AGEs: advanced glycation end-products; AP: Amadori products; BSA: bovine serum albumin; DT: dityrosine; Gal: galactose; GF: glycating factor; Glc: glucose; Fru: fructose; NFK: N-formylkynurenine; PC: protein carbonyls; Rib: ribose; TT: total thiols; ^#^ *p* < 0.05 vs. positive control (GF); ^##^ *p* < 0.01 vs. positive control (GF); ^###^ *p* < 0.001 vs. positive control (GF); ^####^ *p* < 0.0001 vs. positive control (GF); * *p* < 0.05 vs. negative control (BSA); ** *p* < 0.01 vs. negative control (BSA); *** *p* < 0.001 vs. negative control (BSA); **** *p* < 0.0001 vs. negative control (BSA).

The content of protein glycoxidation products (dityrosine and N-formylkynurenine) was considerably higher in samples of BSA with the addition of glucose than in BSA alone. The dityrosine contents were significantly lower in BSA+Glc+aminoguanidine (↓50.1%), BSA+Glc+Trolox (↓45.1%), BSA+Glc+ranitidine (↓87.9%), and BSA+Glc+famotidine (↓11.2%) than in BSA+Glc. The dityrosine content was lower in BSA+Glc+ranitidine (↓78.9%) than in BSA. In turn, the dityrosine contents were higher in BSA+Glc+cimetidine (↑69.9%) and BSA+Glc+famotidine (↑50.8%) than in BSA. The contents of N-formylkynurenine were lower in BSA+Glc+aminoguanidine (↓18.6%), BSA+Glc+Trolox (↓21.5%), BSA+Glc+ranitidine (↓75.4%), and BSA+Glc+famotidine (↓11.3%) than in BSA+Glc. The content of N-formylkynurenine was also considerably lower in BSA+Glc+ranitidine (↓75.6%) and higher in BSA+Glc+aminoguanidine (↑93.6%), BSA+Glc+cimetidine (↑127.4%), BSA+Glc+Trolox (↑87.6%), and BSA+Glc+famotidine (↑110.3%) than in BSA (Figure 2A,B).

The content of protein glycation products (Amadori products and AGEs) was higher after the addition of glucose (BSA+Glc) than in BSA alone. The contents of Amadori products were lower in BSA+Glc+aminoguanidine (↓32.3%), BSA+Glc+Trolox (↓20.4%), BSA+Glc+ranitidine (↓45.9%), and BSA+Glc+famotidine (↓7.2%) than in BSA+Glc. The contents of Amadori products were higher in BSA+Glc+aminoguanidine (↑275.9%), BSA+Glc+Trolox (↑335.2%), BSA+Glc+ranitidine (↑203.6%), BSA+Glc+cimetidine (↑425.9%), and BSA+Glc+famotidine (↑407.6%) than in BSA. The contents of AGEs were lower in BSA+Glc+aminoguanidine (↓29.3%), BSA+Glc+Trolox (↓27.4%), BSA+Glc+ranitidine (↓91.5%), and BSA+Glc+famotidine (↓14.3%) than in BSA+Glc. The contents of AGEs were considerably higher in BSA+Glc+aminoguanidine (↑40.3%), BSA+Glc+Trolox (↑44.9%), BSA+Glc+cimetidine (↑93.6%), and BSA+Glc+famotidine (↑68.2%), and lower in BSA+Glc+ranitidine (↓80.8%) than in BSA (Figure 2C,D). 

The content of PCs was higher after the addition of glucose (BSA+Glc) than in BSA alone. The content of PCs was higher in BSA+Glc+famotidine (↑62.3%) than in BSA+Glc. The contents of PCs were higher in BSA+Glc+cimetidine (↑262.3%) and BSA+Glc+famotidine (↑344.2%) than in BSA. The content of TTs was lower in BSA+Glc+ranitidine (↓51.8%) than in BSA+Glc. In comparison with BSA, the contents of TTs were lower in BSA+Glc and in BSA+Glc after the addition of aminoguanidine, Trolox, ranitidine, cimetidine, and famotidine (↓77.8%, 77.2%, 91.3%, 83.5%, and 85.2%, respectively) (Figure 2E,F).

### 2.3. The Effect of H2 Inhibitors on Protein Glycoxidation, Glycation, and Oxidative Damage in Fructose (Fru)-Induced Albumin Glycation

The contents of protein glycoxidation products (dityrosine and N-formylkynurenine) were considerably higher in BSA with the addition of fructose than in BSA alone. The dityrosine contents were significantly lower in BSA+Fru+aminoguanidine (↓53%), BSA+Fru+Trolox (↓47%), BSA+Fru+ranitidine (↓85%), BSA+Fru+cimetidine (↓50%), and BSA+Fru+famotidine (↓53%) than in BSA+Fru. The dityrosine contents were also significantly lower in BSA+Fru+aminoguanidine (↓21%), BSA+Fru+ranitidine (↓75%), BSA+Fru+cimetidine (↓17%), and BSA+Fru+famotidine (↓21%) than in BSA. The contents of N-formylkynurenine were lower in BSA+Fru+aminoguanidine (↓93%), BSA+Fru+Trolox (↓79%), BSA+Fru+ranitidine (↓86%), BSA+Fru+cimetidine (↓50%), and BSA+Fru+famotidine (↓48%) than in BSA+Fru. The contents of N-formylkynurenine were also considerably lower in BSA+Fru+aminoguanidine (↓84%), BSA+Fru+Trolox (↓50%), and BSA+Fru+ranitidine (↓67%), but higher in BSA+Fru+famotidine (↑21%) than in BSA (Figure 2G,H).

The contents of protein glycation products (Amadori products and AGEs) were also higher after fructose addition (BSA+Fru) than in BSA alone. In turn, the contents of Amadori products were significantly lower in samples of BSA+Fru+aminoguanidine (↓61%), BSA+Fru+Trolox (↓47%), BSA+Fru+ranitidine (↓69%), BSA+Fru+cimetidine (↓49%), and BSA+Fru+famotidine (↓51%) than in BSA+Fru. The contents of Amadori products were higher in BSA+Fru+aminoguanidine (↑116%), BSA+Fru+Trolox (↑194%), BSA+Fru+ranitidine (↑72%), BSA+Fru+cimetidine (↑183%), and BSA+Fru+famotidine (↑172%) than in BSA. The contents of AGEs were considerably higher in BSA+Fru+ranitidine (↑96%), BSA+Fru+cimetidine (↑164%), and BSA+Fru+famotidine (↑138%) than in BSA (Figure 2I,J). 

The content of PCs was higher in the presence of fructose (BSA+Fru) than in BSA alone (↑177%), and lower in BSA+Fru+ranitidine (↓72%) than in BSA+Fru. The contents of TTs were lower in BSA with the addition of fructose (BSA+Fru), aminoguanidine, Trolox, ranitidine, cimetidine, and famotidine (↓81%, 52%, 45%, 62%, 64%, and 72%, respectively) than in BSA alone. In comparison with BSA+Fru, the TT contents increased after the addition of aminoguanidine, Trolox, ranitidine, cimetidine, and famotidine (↑185%, 202%, 54%, 99%, and 57%, respectively) (Figure 2K,L).

### 2.4. The Effect of H2 Inhibitors on Protein Glycoxidation, Glycation, and Oxidative Damage in Galactose (Gal)-Induced Albumin Glycation

The contents of protein glycoxidation products (dityrosine and N-formylkynurenine) were considerably higher in BSA samples with the addition of galactose than in BSA alone. The dityrosine contents were lower in BSA+Gal+aminoguanidine (↓24.2%), BSA+Gal+Trolox (↓29.9%), BSA+Gal+ranitidine (↓92.6%), and BSA+Gal+famotidine (↓11.5%) than in BSA+Gal. The dityrosine contents were lower in BSA+Gal+ranitidine (↓87.5%) and higher in BSA+Gal+aminoguanidine (↑27.2%), BSA+Gal+Trolox (↑17.6%), BSA+Gal+cimetidine (↑68.7%), and BSA+Gal+famotidine (↑48.7%) than in BSA. The contents of N-formylkynurenine were lower in BSA+Gal+aminoguanidine (↓21.9%), BSA+Gal+Trolox (↓34.8%), and BSA+Gal+ranitidine (↓86.2%) than in BSA+Gal. The contents of N-formylkynurenine were lower in BSA+Gal+ranitidine (↓67.5%) and higher in BSA+Gal+aminoguanidine (↑83.6%), BSA+Gal+Trolox (↑53.4%), BSA+Gal+cimetidine (↑150.6%), and BSA+Gal+famotidine (↑122.6%) than in BSA (Figure 2M,N).

The contents of protein glycation products (Amadori products, AGEs) were higher after the addition of galactose (BSA+Gal) than in BSA alone. The contents of Amadori products were lower in BSA+Gal+aminoguanidine (↓23.9%), BSA+Gal+Trolox (↓14.9%), and BSA+Gal+ranitidine (↓35.5%) than in BSA+Gal. The contents of Amadori products were higher in BSA+Gal+aminoguanidine (↑314.6%), BSA+Gal+Trolox (↑363%), BSA+Gal+ranitidine (↑251%), BSA+Gal+cimetidine (↑451.7%), and BSA+Gal+famotidine (↑453.9%) than in BSA. The contents of AGEs were lower in BSA+Gal+aminoguanidine (↓24.8%), BSA+Gal+Trolox (↓27.9%), BSA+Gal+ranitidine (↓92.3%), and BSA+Gal+famotidine (↓12.1%) than in BSA+Gal. The contents of AGEs were lower in BSA+Gal+ranitidine (↓84.8%) and higher in BSA+Gal+aminoguanidine (↑47.5%), BSA+Gal+Trolox (↑41.5%), BSA+Gal+cimetidine (↑103.3%), and BSA+Gal+famotidine (↑72.4%) than in BSA (Figure 2O,P)

The content of PCs was higher after the addition of galactose (BSA+Gal) than in BSA alone. The contents of PCs were lower in BSA+Gal+Trolox (↓56.9%), BSA+Gal+ranitidine (↓34.1%), and BSA+Gal+cimetidine (↓34.5%) than in BSA+Gal. The contents of PCs were higher in BSA+Gal+aminoguanidine (↑114.4%) and BSA+Gal+famotidine (↑111.3%) than in BSA. The contents of TTs were higher in BSA+Gal+aminoguanidine (↑140.9%) and BSA+Gal+Trolox (↑112.1%) than in BSA+Gal. In comparison with BSA, the contents of TTs were lower in BSA+Gal and in BSA+Gal with the addition of aminoguanidine, Trolox, ranitidine, cimetidine, and famotidine (↓55.9%, 61.2%, 85.9%, 84%, and 83.2%, respectively) (Figure 2Q,R).

### 2.5. The Effect of H2 Inhibitors on Protein Glycoxidation, Glycation, and Oxidative Damage in Ribose (Rib)-Induced Albumin Glycation 

The contents of protein glycoxidation products (dityrosine and N-formylkynurenine) were considerably higher in BSA samples with the addition of ribose than in BSA alone. The dityrosine contents were lower in BSA+Rib+aminoguanidine (↓53.3%), BSA+Rib+Trolox (↓47.8%), BSA+Rib+ranitidine (↓69.8%), and BSA+Rib+famotidine (↓13.2%) than in BSA+Rib. The dityrosine contents were lower in BSA+Rib+aminoguanidine (↓20.9%) and BSA+Rib+ranitidine (↓47.8%) than in BSA. In turn, the dityrosine contents were higher in BSA+Rib+cimetidine (↑61.9%) and BSA+Rib+famotidine (↑46.3%) than in BSA. The contents of N-formylkynurenine were lower in BSA+Rib+aminoguanidine (↓83.7%), BSA+Rib+Trolox (↓79.2%), BSA+Rib+ranitidine (↓64.4%), and BSA+Rib+famotidine (↓14.8%) than in BSA+Rib. The contents of N-formylkynurenine were considerably lower in BSA+Rib+aminoguanidine (↓83.4%), BSA+Rib+Trolox (↓50.2%), and BSA+Rib+ranitidine (↓15%), and higher in BSA+Rib+cimetidine (↑125.4%) and BSA+Rib+famotidine (↑103.5%) than in BSA (Figure 2S,T).

The content of protein glycation products (Amadori products and AGEs) was higher after the addition of ribose (BSA+Rib) than in BSA alone. The contents of Amadori products were lower in BSA+Rib+aminoguanidine (↓60.7%), BSA+Rib+Trolox (↓46.1%), and BSA+Rib+ranitidine (↓38%) than in BSA+Rib. The contents of Amadori products were higher in BSA+Rib+aminoguanidine (↑114%), BSA+Rib+Trolox (↑193.4%), BSA+Rib+ranitidine (↑237.2%), BSA+Rib+cimetidine (↑424.6%), and BSA+Rib+famotidine (↑431.8%) than in BSA. The contents of AGEs were lower in BSA+Rib+aminoguanidine (↓42%), BSA+Rib+Trolox (↓23.2%), BSA+Rib+ranitidine (↓67.5%), and BSA+Rib+famotidine (↓13.7%) than in BSA+Rib. The contents of AGEs were considerably higher in BSA+Rib+aminoguanidine (↑15%), BSA+Rib+Trolox (↑56.7%), BSA+Rib+cimetidine (↑90.5%), and BSA+Rib+famotidine (↑69.4%), and lower in BSA+Rib+ranitidine (↓36.3%) than in BSA (Figure 2U,V). 

The contents of PCs were higher in BSA with the addition of ribose (BSA+Rib) than in BSA alone. The contents of PCs were lower in BSA+Rib+aminoguanidine (↓47%) and BSA+Rib+Trolox (↓37.6%) than in BSA+Rib. The contents of PCs were higher in BSA+Rib+ranitidine (↑147.8%), BSA+Rib+cimetidine (↑138.9%), and BSA+Rib+famotidine (↑164.2%) than in BSA. The contents of TTs were higher in BSA+Rib+aminoguanidine (↑185%) and BSA+Rib+Trolox (↑202.2%) than in BSA+Rib. The content of TTs was lower in BSA+Rib than in BSA. In comparison with BSA, the content of TTs decreased after the addition of aminoguanidine, Trolox, ranitidine, cimetidine, and famotidine to BSA+Rib (↓47.9%, 44.7%, 83.2%, 82.2%, and 81%, respectively) (Figure 2W,X). 

### 2.6. The Effect of H2 Inhibitors on Protein Glycoxidation, Glycation, and Oxidative Damage in Glyoxal (GO)-Induced Albumin Glycation 

**Figure 3:**
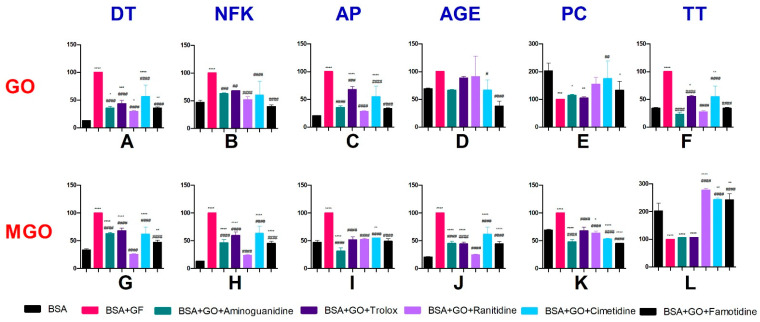
The effects of H2 inhibitors on protein glycoxidation (**A**,**B**,**G**,**H**), glycation (**C**,**D**,**I**,**J**), and oxidative damage (**E**,**F**,**K**,**L**) in aldehyde-induced albumin glycation. AGEs: advanced glycation end-products; AP: Amadori products; BSA: bovine serum albumin; DT: dityrosine; GF: glycating factor; GO: glyoxal; MGO: methylglyoxal; NFK: N-formylkynurenine; PC: protein carbonyls; TT: total thiols; ^#^ *p* < 0.05 vs. positive control (GF); ^##^ *p* < 0.01 vs. positive control (GF); ^###^ *p* < 0.001 vs. positive control (GF); ^####^ *p* < 0.0001 vs. positive control (GF); * *p* < 0.05 vs. negative control (BSA); ** *p* < 0.01 vs. negative control (BSA); *** *p* < 0.001 vs. negative control (BSA); **** *p* < 0.0001 vs. negative control (BSA).

The content of protein glycoxidation products (dityrosine and N-formylkynurenine) was considerably higher in BSA samples with the addition of glyoxal than in BSA alone. The dityrosine contents were lower in BSA+GO+aminoguanidine (↓77.3%), BSA+GO+Trolox (↓44.9%), BSA+GO+ranitidine (↓72.7%), BSA+GO+cimetidine (↓45.1%), and BSA+GO+famotidine (↓66%) than in BSA+GO. The dityrosine contents were higher in BSA+GO+cimetidine (↑62.2%) and BSA+GO+Trolox (↑62.6%) than in BSA. The contents of N-formylkynurenine were lower in BSA+GO+aminoguanidine (↓64.4%), BSA+GO+Trolox (↓56.6%), BSA+GO+ranitidine (↓70.4%), BSA+GO+cimetidine (↓43.7%), and BSA+GO+famotidine (↓64.5%) than in BSA+GO. The contents of N-formylkynurenine were higher in BSA+GO+aminoguanidine (↑176.5%), BSA+GO+Trolox (↑236.9%), BSA+GO+ranitidine (↑130.2%), BSA+GO+cimetidine (↑337.6%), and BSA+GO+famotidine (↑176.1%) than in BSA (Figure 3A,B).

The content of protein glycation products (Amadori products, AGEs) was higher after the addition of glyoxal (BSA+GO) than in BSA alone. The contents of Amadori products were lower in BSA+GO+aminoguanidine (↓37.3%), BSA+GO+Trolox (↓31.9%), BSA+GO+ranitidine (↓48%), BSA+GO+cimetidine (↓39.9%), and BSA+GO+famotidine (↓60.5%) than in BSA+GO. The contents of AGEs were lower in BSA+GO+aminoguanidine (↓64.9%), BSA+GO+Trolox (↓32.2%), BSA+GO+ranitidine (↓72%), BSA+GO+cimetidine (↓45.2%), and BSA+GO+famotidine (↓67%) than in BSA+GO. The contents of AGEs were considerably higher in BSA+GO+Trolox (↑230.7%) and BSA+GO+cimetidine (↑167.1%) than in BSA (Figure 3C,D). 

The content of PCs increased after the addition of glyoxal (BSA+GO) in comparison with BSA alone. The contents of PCs were lower in BSA+GO+cimetidine (↓33.1%) and BSA+GO+famotidine (↓62.1%) than in BSA+GO. The content of TTs was higher in BSA+GO+cimetidine (↑75.2%) than in BSA+GO. In comparison with BSA, the contents of TTs decreased in BSA+GO and in BSA+GO after the addition of aminoguanidine, Trolox, and famotidine (↓43.5%, 48.4%, and 34.5%, respectively) (Figure 3E,F).

### 2.7. The Effect of H2 Inhibitors on Protein Glycoxidation, Glycation, and Oxidative Damage in Methylglyoxal (MGO)-Induced Albumin Glycation 

The content of protein glycoxidation products (dityrosine and N-formylkynurenine) was considerably higher in BSA samples with the addition of methylglyoxal than in BSA alone. The dityrosine contents were lower in BSA+MGO+aminoguanidine (↓37.2%), BSA+MGO+Trolox (↓31.9%), BSA+MGO+ranitidine (↓74.7%), BSA+MGO+cimetidine (↓37.7%), and BSA+MGO+famotidine (↓53%) than in BSA+MGO. The dityrosine contents were higher in BSA+MGO+aminoguanidine (↑87.7%), BSA+MGO+Trolox (↑103.6%), BSA+MGO+cimetidine (↑86.1%), and BSA+MGO+famotidine (↑40.5%) than in BSA. The contents of N-formylkynurenine were lower in BSA+MGO+aminoguanidine (↓53.7%), BSA+MGO+Trolox (↓40.3%), BSA+MGO+ranitidine (↓76.6%), BSA+MGO+cimetidine (↓36.8%), and BSA+MGO+famotidine (↓54.9%) than in BSA+MGO. The contents of N-formylkynurenine were higher in BSA+MGO+aminoguanidine (↑260.1%), BSA+MGO+Trolox (↑363.8%), BSA+MGO+cimetidine (↑391.5%), and BSA+MGO+famotidine (↑250.4%) than in BSA (Figure 3G,H).

The content of protein glycation products (Amadori products and AGEs) increased after the addition of methylglyoxal (BSA+MGO) relative to BSA alone. The contents of Amadori products were lower in BSA+MGO+aminoguanidine (↓68.8%), BSA+MGO+Trolox (↓48.4%), BSA+MGO+ranitidine (↓47.4%) BSA+MGO+cimetidine (↓45.1%), and BSA+MGO+famotidine (↓50.9%) than in BSA+MGO. The contents of Amadori products were lower in BSA+MGO+aminoguanidine (↓33.7%) and higher in BSA+MGO+cimetidine (↑16.6%) than in BSA. The contents of AGEs were lower in BSA+MGO+aminoguanidine (↓55%), BSA+MGO+Trolox (↓55.9%), BSA+MGO+ranitidine (↓75.6%), BSA+MGO+cimetidine (↓38.3%), and BSA+MGO+famotidine (↓55.4%) than in BSA+MGO. The contents of AGEs were higher in BSA+MGO+aminoguanidine (↑119.6%), BSA+MGO+Trolox (↑115%), BSA+MGO+cimetidine (↑201%), and BSA+MGO+famotidine (↑117.7%) than in BSA (Figure 3I,J).

The content of PCs increased after the addition of methylglyoxal (BSA+MGO) relative to BSA alone. The contents of PCs were lower in BSA+MGO+aminoguanidine (↓52%), BSA+MGO+Trolox (↓32.1%), BSA+MGO+ranitidine (↓36.2%) BSA+MGO+cimetidine (↓46.8%), and BSA+MGO+famotidine (↓54.9%) than in BSA+MGO. The contents of PCs decreased in BSA+MGO+aminoguanidine (↓30.6%), BSA+MGO+ranitidine (↓7.7%), BSA+MGO+cimetidine (↓23%), and BSA+MGO+famotidine (↓34.7%) relative to BSA. The contents of TTs were higher in BSA+MGO+ranitidine (↑177.4%), BSA+MGO+cimetidine (↑143.8%), and BSA+MGO+famotidine (↑143%) than in BSA+MGO. In comparison with BSA, the contents of TTs decreased in BSA+MGO and in BSA+MGO with the addition of aminoguanidine and Trolox (↓47.9% and 47.3%, respectively). The contents of TTs were higher in BSA+MGO+ranitidine (↑36.9%), BSA+MGO+cimetidine (↑20.4%), and BSA+MGO+famotidine (↑20%) than in BSA (Figure 3K,L).

### 2.8. Antioxidant Activity of H2 Inhibitors

As the anti-glycation properties of H2 inhibitors may be due to their antioxidant properties, their ability to scavenge free radicals and bind transition metal ions was also assessed.

**Figure 4:**
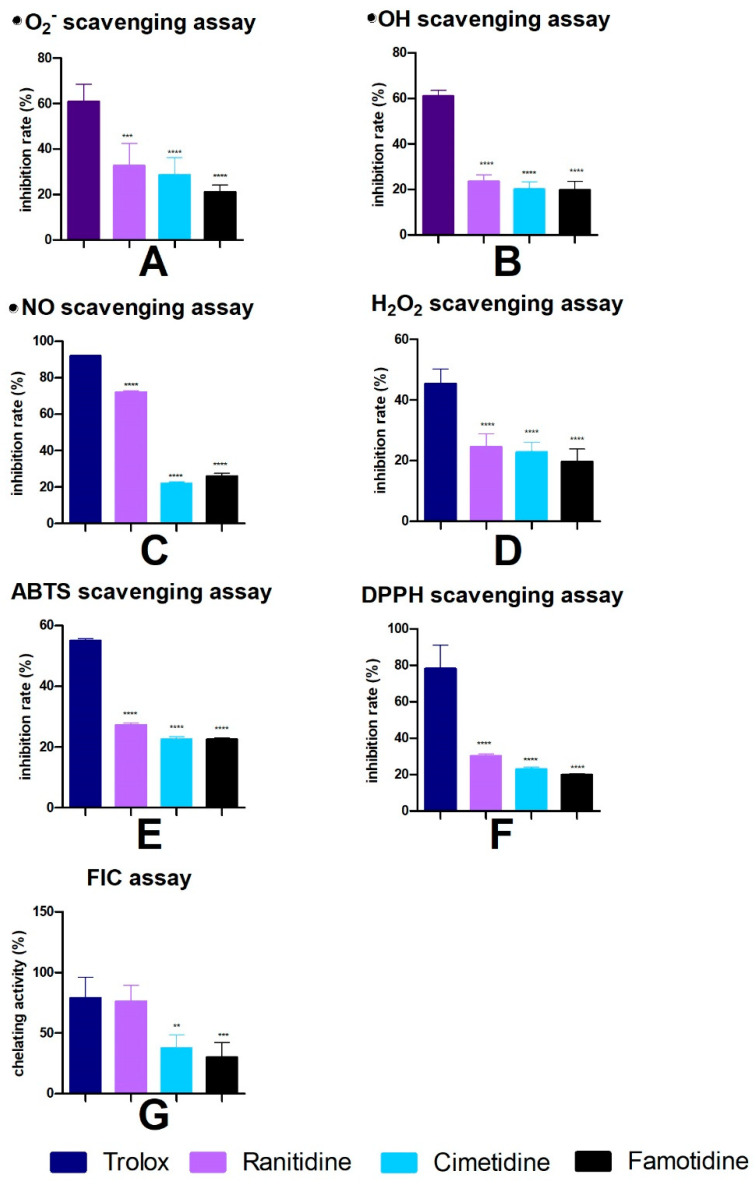
The effect of H2 inhibitors on superoxide anions (^•^O_2_^−^, **A**), hydroxyl radicals (^•^OH, **B**), nitric oxide (^•^NO, **C**), hydrogen peroxide (H_2_O_2_, **D**), 2,2’-azino-bis(3-ethylbenzothiazoline)-6-sulfonic acid (ABTS, **E**) and 2,2-diphenyl-1-picrylhydrazyl radical (DPPH, **F**) scavenging activity, as well as ferrous iron chelating (FIC, **G**). ** *p* < 0.01 vs. positive control (Trolox); *** *p* < 0.001 vs. positive control (Trolox); **** *p* < 0.0001 vs. positive control (Trolox).

At the concentration tested (1 mM), H2 blockers had a scavenging capacity of 20–70% for superoxide anions (^•^O_2_^−^), hydroxyl radicals (^•^OH), nitric oxide (^•^NO), hydrogen peroxide (H_2_O_2_), 2,2’-azino-bis(3-ethylbenzothiazoline)-6-sulfonic acid (ABTS), and 2,2-diphenyl-1-picrylhydrazyl (DPPH) radicals. The strongest antioxidant properties were found in ranitidine, which additionally had an ability to bind transition metal ions. The iron chelating capacity of ranitidine was no different to that of Trolox, which is a potent model antioxidant (Figure 4).

### 2.9. Molecular Docking Analysis

The molecular docking analysis in AutoDock Vina revealed that H2 blockers had a low affinity for BSA (below 5 kcal/mol) (Table S2). Ranitidine exhibited the lowest affinity for BSA in the tested group of drugs. In two binding sites, the root-mean-square deviation (RMSD) was below 3, and in one hypothetical binding site (binding mode 1), a polar bond was identified between ranitidine and the side chain of thyrosine-161 in BSA (Figure S2). 

## 3. Discussion

H2 receptor antagonists are a group of drugs that inhibit gastric juice secretion in gastrointestinal diseases [12]. However, there is evidence to suggest that H2 blockers have a broader spectrum of activity and can potentially deliver antioxidant effects [21,22]. Cimetidine inhibits lipid peroxidation in the gastric mucosa [33] and minimizes liver damage caused by the excessive intake of alcohol [34]. Ranitidine exerts indirect antioxidant effects by suppressing TNF-α in LPS-stimulated monocytes [25]. However, the antioxidant properties of H2 blockers have not been fully elucidated, and their anti-glycation potential has not been studied to date. Therefore, the present study was undertaken to determine the impact of H2 antagonists on protein glycoxidation in vitro. In the analyzed group of drugs, ranitidine was the only H2 blocker that significantly inhibited BSA glycation in all tested models. The anti-glycation potential of ranitidine is comparable to that of aminoguanidine. 

Bovine serum albumin and human albumin have a highly similar structure [35]. Bovine serum albumin contains 35 thiol groups, 34 of which are linked by disulfide bridges. The free thiol group in albumin oxidizes and binds various ligands [36]. The thiol group in Cys is a strong nucleophile that is easily glycated with sugar molecules to produce S-carboxymethyl-l-cysteine (CMC) [37]. Cross-links are also formed between proteins when thiols react with sugars. As a result, in the present study, the content of TTs decreased in BSA samples with the addition of all glycating sugars. The content of AGEs, (irreversible products that are generated in the late stage of glycation from Amadori products in the Maillard reaction) also increased in these samples. Glycoxidation products are highly heterogeneous, which is why the fluorescence of protein glycoxidation products (dityrosine and N-formylkynurenine) and the concentrations of PCs were also analyzed. In hyperglycemia, the auto-oxidation of reducing sugars leads to ROS overproduction, which contributes to protein oxidation. Therefore, the biomarkers of BSA glycoxidation (↑dityrosine and ↑N-formylkynurenine), glycation (↑Amadori products and ↑AGEs), and oxidation (↑PCs and ↓TTs) had to be evaluated to confirm the presence of carbonyl stress under exposure to the applied glycating agents. Bovine serum albumin is also glycated by dicarbonyl derivatives (GO and MGO), which are the direct precursors of Amadori products and AGEs [31,38].

In the group of the tested H2 blockers, only ranitidine considerably decreased the BSA glycoxidation rate. In most of the analyzed models, the contents of PCs (↓PCs), protein glycoxidation products (↓dityrosine and ↓N-formylkynurenine), and early (↓Amadori products) and late-stage (↓AGEs) protein glycation products decreased in samples of glycated BSA with the addition of ranitidine relative to BSA with the addition of the glycating agents (positive control). Ranitidine also increased the TT concentration in Fru- and MGO-induced models of BSA glycation. Interestingly, in some cases, ranitidine decreased the rate of protein glycoxidation below the level noted in BSA samples without the addition of a glycating agent (negative control) (↓dityrosine in Glc; ↓AGE in Glc; ↓dityrosine in Fru; ↓N-formylkynurenine in Fru; ↓dityrosine in Gal; ↓N-formylkynurenine in Gal; ↓AGE in Gal; ↓dityrosine in Rib; ↓N-formylkynurenine in Rib; ↓AGE in Rib; and ↓dityrosine in MGO). 

To ensure objectivity, the pharmacological potential of ranitidine was compared with that of reference substances with proven antioxidant (Trolox) and anti-glycation properties (aminoguanidine) [39,40]. At the tested concentration (1 mM), ranitidine was as effective or more effective than Trolox and aminoguanidine in inhibiting BSA glycoxidation (↓AGE in Glc; ↓dityrosine in Fru; ↓PCs in Fru; ↓dityrosine in Gal; ↓N-formylkynurenine in Gal; ↓Amadori products in Gal; ↓AGE in Gal; ↓dityrosine in Rib; ↓dityrosine in MGO; and ↓AGE in MGO). Trolox is a water-soluble analog of α-tocopherol with high anti-radical activity, and it is widely used as a reference compound in assessments of antioxidant properties [40]. Aminoguanidine is the most potent anti-glycation agent described to date [41]. Aminoguanidine contains hydrazine which reacts with dicarbonyl compounds to produce 3-amino-1,2,4-triazines. These compounds do not form cross-links, and they are unable to further bind to proteins. However, aminoguanidine is strongly cytotoxic; it did not pass animal tests and has not been approved for medicinal use [42]. Unlike aminoguanidine, H2 antagonists are generally well-tolerated [13]. Adverse reactions, mainly hypotension, headache, dizziness, fatigue, diarrhea, constipation, rash, gynecomastia in men, loss of libido, and impotence, are rarely reported. Importantly, cytotoxic effects, such as anemia, leukopenia, and agranulocytosis, are very rarely reported [43,44,45].

Three groups of substances have anti-glycation properties: (1) antioxidants, (2) compounds that break AGE-derived protein crosslinks (AGE-breakers), and (3) compounds that compete with protein amino groups for the addition of carbonyl groups. Therefore, the anti-glycation effects of H2 receptor antagonists, observed in this study, could be attributed to their antioxidant properties. This was confirmed by the results of our research. The tested H2 blockers had the ability to scavenge ^•^O_2_^−^, ^•^OH, ^•^NO, and H_2_O_2_, but also the synthetic radicals ABTS^•^ and DPPH^•^. Interestingly, the strongest antioxidant properties were found in ranitidine, which additionally had the ability to bind transition metal ions (↑FIC). The systematic literature review (Table S1) also revealed that ranitidine and cimetidine are potent scavengers of ^•^OH which are produced when Fe^2+^ reacts with H_2_O_2_ (Fenton reaction) [24,46,47,48]. In other studies, cimetidine and ranitidine reacted with ^•^O_2_^−^ radicals [34,49]. Ranitidine was found to be the most potent scavenger of DPPH radicals among all tested H2 blockers [19]. Ranitidine also exhibited indirect antioxidant activity by suppressing neutrophil activation (decreased production of neutrophil elastase and ^•^O_2_^−^) in vivo [22]. Ranitidine could also compete with protein amino groups for the addition of carbonyl groups. In the group of the tested H2 blockers, ranitidine was characterized by the lowest binding energy for BSA sites in the molecular docking analysis. In two binding sites, the RMSD was below 3, and a polar bond between ranitidine and the side chain of thyrosine-161 in BSA was identified in one binding site.

The anti-glycoxidative activity of ranitidine could be attributed to its molecular structure, although the present study did not provide evidence for the above. The presence of a dimethylaminomethyl group in five-member heterocyclic rings increases a drug’s alkalinity [50]. The heterocyclic ring and the polar part (1-N’-methyl-2-nitroethane-1,1-diamine) are linked by a bond with thioether structure (thioethyl methylene), whereas the presence of a nitro functional group increases lipophilicity and antihistamine activity [51]. However, ranitidine is a furan derivative. Various furan derivatives with antioxidant, anti-inflammatory, antiviral, and antiproliferative properties have been described in the literature [52,53,54]. Furan derivatives (including natural benzofurans, furan fatty acids, agarofurans, and furanocoumarins) have broad-spectrum pharmacological activity (such as scavenging ^•^OH radicals and chelating transition metal ions), and they inhibit the activation of the NF-κB, mitogen-activated protein kinase (MAPK), and peroxisome proliferator-activated receptor-gamma (PPAR-ɣ) pathways in vivo [55]. Therefore, further in vitro and in silico studies are needed to assess the extent to which the biological activity of H2 receptor blockers is influenced by their structure.

H2 receptor antagonists have been used globally for more than four decades. Many gastrointestinal diseases intensify oxidative and carbonyl stress, and H2 blockers with potential antioxidant/anti-glycation activity can be helpful in optimizing pharmacotherapy. This is the first study to describe the anti-glycoxidant properties of ranitidine. Ranitidine is as effective as aminoguanidine and Trolox in inhibiting BSA glycation and oxidation, which is why further research is needed, particularly in patients with diseases that promote protein glycation (diabetes, cardiovascular, and neurodegenerative diseases). The anti-glycation properties of ranitidine may be due to its antioxidant activity and its ability to bind transition metal ions. The systematic literature review also revealed that H2 receptor blockers modulate redox homeostasis, carbonyl stress, and the inflammatory response in vivo, which also implies that further studies are recommended.

Finally, the limitations of this study and the next steps should be discussed. In this experiment, we used sodium azide (as a preservative), which may have pro-oxidant properties. However, at the concentration used, the compound does not show this activity [56]. The effect of sodium azide was identical for all samples, and its addition was determined by kinetic studies of BSA glycation [57,58,59]. The present study cannot replace animal tests or clinical trials, and the anti-glycoxidant effects of H2 blockers should also be examined in vivo. It should be noted that ranitidine is biotransformed in the body, and the produced metabolites can also exhibit anti-glycoxidant potential [60,61] The major metabolite is ranitidine N-oxide, and ranitidine S-oxide and desmethyl ranitidine are produced in smaller quantities. Therefore, further research is also needed to examine ranitidine metabolites.

## 4. Materials and Methods

### 4.1. Systematic Review

The literature review was conducted from the PubMed database, and it involved articles published between 1986 and 2023. The following keywords were used: ranitidine and antioxidant, ranitidine and oxidative stress, ranitidine and glycation, ranitidine and carbonyl stress, ranitidine and advanced glycation protein products, cimetidine and antioxidant, cimetidine and oxidative stress, ranitidine and ROS, cimetidine and ROS, famotidine and ROS, cimetidine and glycation, cimetidine and carbonyl stress, cimetidine and advanced glycation protein products, famotidine and antioxidant, famotidine and oxidative stress, famotidine and glycation, famotidine and carbonyl stress, and famotidine and advanced glycation protein products. The inclusion and exclusion criteria are presented in Table 1.

### 4.2. Reagents and Equipment

All reagents for the study were supplied by Sigma-Aldrich (Nümbrecht, Germany/Saint Louis, MO, USA). Before the study, all solutions were passed through syringe member filters with a diameter of 0.22 mm (Biosens, Warsaw, Poland).

Absorption/fluorescence spectra were measured with the Infinite M200 PRO multimode plate reader (Tecan Group Ltd., Männedorf, Switzerland).

### 4.3. Bovine Serum Albumin (BSA)

Bovine serum albumin was glycated using previously described methods [29,30,62,63,64]. BSA (purity > 98%; molar mass—66,430 Da) was dissolved in a sodium phosphate buffer (0.1 M, pH 7.4) containing 0.2% sodium azide (as a preservative). Glycation was induced by the use of the following sugars: glucose (Glc), fructose (Fru), galactose (Ga), and ribose (Rib), as well as the following aldehydes: glyoxal (GO) and methylglyoxal (MGO). The concentrations of sugars and aldehydes at which glycation was initiated and the optimal incubation conditions were determined/validated based on the results of a previous kinetic study. The described experimental design is routinely applied to determine the anti-glycation and antioxidant properties of new substances. Despite the fact that the sugar, aldehyde, and oxidant concentrations significantly exceeded physiological levels, they were convenient for the rapid modeling of processes that occur in the human body over weeks or months. To determine the influence of H2 blockers on protein glycoxidation, BSA was incubated with 0.5 M of sugars for 6 days or with 2.5 mM of aldehydes for 12 h in the dark in a shaking incubator (50 rpm; 37 °C) [29,63,64,65]. The concentration of BSA in the final solutions was 0.09 mM. Two reference substances were used to compare the results for ranitidine, cimetidine, and famotidine: aminoguanidine (a popular inhibitor of protein glycation) and Trolox (inhibitor of protein oxidation). Each substance was applied at a concentration of 1 mM based on the results of in vitro kinetic studies, proportionally to the high concentrations of glycation agents [63,64]. This study involved three independent experiments with two replicates each.

#### 4.3.1. Protein Glycoxidation Products: Dityrosine (DT) and N-formylkynurenine (NFK)

Dityrosine (DT) and N-formylkynurenine (NFK) were identified by measuring fluorescence emission and excitation spectra at wavelengths of 325/434 nm and 330/415 nm. The samples were diluted with 0.1 M sulfuric acid (1:5, *v*:*v*) before the measurements. The results were standardized to the fluorescence of 0.1 mg/mL of quinine sulfate in 0.1 M sulfuric acid [66].

#### 4.3.2. Protein Glycation Products: Amadori products and Advanced Glycation End-Products (AGE)

The content of Amadori products was determined in a colorimetric analysis with the use of nitro blue tetrazolium (NBT). Absorbance was measured at a wavelength of 525 nm based on the molar extinction coefficient for monoformazan (12,640 M^−1^ cm^−1^) [32].

The content of AGEs was determined with the use of a spectrofluorometer. The samples were diluted with PBS (1:5, *v*:*v*) before readout. AGE-specific fluorescence was measured at a wavelength of 440/370 nm in a 96-well microplate reader.

#### 4.3.3. Protein Oxidation Products: Protein Carbonyls (PCs) and Total Thiols (TTs)

The reaction between carbonyls and 2,4-dinitrophenylhydrazine (2,4-DNPH) in oxidatively damaged proteins was used to determine the content of protein carbonyls (PCs). The absorbance of reaction products was measured in a colorimetric assay at 355 nm with the use of the absorption coefficient for 2,4-DNPH (22,000 M^−1^ cm^−1^) [67].

Total thiols (TTs) were quantified with the use of a spectrophotometer and Ellman’s reagent in 0.1 M of phosphate buffer. The content of TTs was read from a standard curve for reduced glutathione (GSH) [68].

### 4.4. Antioxidant Activity of H2 Inhibitors

The antioxidant properties of ranitidine, cimetidine, and famotidine solutions (1 mM) were also assessed by evaluating their effects on free radical scavenging and transition metal binding.

#### 4.4.1. ^•^O_2_^−^ Scavenging Assay

The experimental procedure involved mixing x μL of the sample solution with (2950 − x) μL of Tris-HCl buffer (0.05 M, pH 7.4, 37 °C), which also contained 1 mM Na_2_EDTA and 50 μL of pyrogallol (60 mM in 1 mM HCl, 37 °C). The mixture was then vigorously shaken by hand at 37 °C. The absorbance at a wavelength of 325 nm was measured against the Tris-HCl buffer every 30 s for a duration of 5 min. These measurements were taken at pH 7.4 [69].

#### 4.4.2. •OH Scavenging Assay

The scavenging activity of hydroxyl radicals (•OH) was determined using a modified method based on Su et al. [70]. The reaction mixture contained ferrous sulfate (8 mM), hydrogen peroxide (6 mM), distilled water (0.5 mL), samples (1.0 mL), and sodium salicylate (20 mM) in a total volume of 2.0 mL. The mixture was incubated at 37 °C for 1 h and the absorbance was measured at 562 nm.

#### 4.4.3. •NO Scavenging Assay

The nitric oxide (•NO) scavenging activity of the ranitidine, cimetidine, and famotidine samples was measured using sodium nitroprusside as the NO donor following the method described by Nitha et al. [71] In brief, a volume of 0.1 mL of the sample solution was mixed with 0.2 mL of phosphate-buffered saline containing sodium nitroprusside (5 mM) and incubated at 25 °C for 150 min. Then, 0.5 mL of the reaction mixture was removed and mixed with 0.5 mL of Griess reagent. The absorbance was measured at 546 nm and the inhibition of NO generation was estimated by comparing the absorbance values with that of the control without the sample solution.

#### 4.4.4. H_2_O_2_ Scavenging Assay

The scavenging activity of H_2_O_2_ was measured using the ferrous ion oxidation xylenol orange (FOX) reagent, which was prepared by combining butylated hydroxytoluene (BHT), sulfuric acid, xylenol orange, and ammonium ferrous sulfate in a 90% methanol–water solution. The samples were mixed with 50 mM H_2_O_2_ (final concentration: 0.2 mg/mL) and incubated for 30 min at room temperature. The H_2_O_2_ sample solution was then mixed with HPLC-grade methanol and FOX reagent, and the mixture was incubated for another 30 min. The absorbance of the ferric–xylenol orange complex was measured at 560 nm [72].

#### 4.4.5. 2,2’-Azino-bis(3-ethylbenzothiazoline)-6-Sulfonic Acid (ABTS) Scavenging Assay

The 2,2’-azino-bis(3-ethylbenzothiazoline)-6-sulfonic acid (ABTS) assay was performed following a modified protocol of Wang et al. [73]. In summary, 10 μL of the diluted sample was added to 290 μL of an ABTS solution containing 2 mM ABTS diammonium salt and 3.5 mM potassium persulfate, followed by the addition of methanol to a final volume of 300 μL. A control sample containing only the ABTS solution was also prepared. The mixture was incubated for 10 min and the absorbance was measured at 750 nm.

#### 4.4.6. 2,2-Diphenyl-1-picrylhydrazyl Radical (DPPH) Scavenging Assay

The free radicals scavenging activity was assessed using the Brand–Williams [74] method. To initiate the reaction, 390 µL of a methanolic dilution of 2,2-diphenyl-1-picrylhydrazyl radical (DPPH) was combined with 10 µL of each sample (ranitidine, cimetidine, and famotidine), and the mixture was carefully transferred to a 96-well microplate. The microplate was then placed in a dark environment at room temperature for a duration of 30 min, allowing the reaction to take place undisturbed. Following this incubation period, the absorbance of the reaction mixture was measured at a wavelength of 515 nm.

#### 4.4.7. Ferric Ion Chelating (FIC) Assay

The ferric ion chelating (FIC) assay, as described by Hsu et al. [75], was utilized to determine the effects of ranitidine, cimetidine, and famotidine samples on ferrous chloride. In brief, 0.5 mL of the aforementioned samples were mixed with 100 μL of ferrous chloride (0.6 mM) and 0.9 mL of methanol. After 10 min at room temperature, 0.1 mL of ferrozine solution (5 mM) was added and the reaction mixture was left at room temperature for an additional 5 min. The absorbance of the mixture was determined at 562 nm, and the FIC effect (%) was calculated from the decrease in absorbance compared with the control.

### 4.5. Molecular Docking

Molecular docking is a computational technique for investigating the interaction between a ligand (often a drug molecule) and a macromolecule (usually a protein). The goal is to predict the ligand’s optimal binding mode and affinity to the macromolecule, which can provide valuable information for the discovery and development of new drugs [76]. The present study focused on the interaction between three drugs (ranitidine, cimetidine, and famotidine) and the BSA protein. The X-ray crystal structure of BSA with a resolution of 2.47 Å was obtained from the Protein Data Bank (PDB ID: 4F5S) to provide a highly detailed model of the protein’s structure. BSA and the analyzed drugs were prepared for docking simulations with the use of AutoDock MGL Tools software. Water molecules were removed from the protein structure, and polar hydrogens and Kollman’s partial charges were added to protein and drug molecules. The protein structure was saved in the PDBQT format, which is compatible with AutoDock Vina software, a popular program for docking simulations. Docking simulations were performed in AutoDock Vina with a 40 × 40 × 40 grid box with 0.375 Å spacing, centered at coordinates 9.324, −23.335, and 5.687. The exhaustiveness value, which determines the number of binding poses sampled during a docking simulation, was set to 8. A higher exhaustiveness value increases computational time, but it can produce more accurate predictions of binding modes and affinities. The docking results were analyzed using PyMOL 2.5 software to provide a visual representation of protein and drug molecules and their interactions. The optimal binding modes of each drug were identified, and these findings can be used to inform the development of more effective drug therapies in the future [38,65].

### 4.6. Statistical Analysis

The results were expressed as the percentage of control samples (BSA with the addition of glycating factors). The statistical significance of the results was determined by one-way analysis of variance (ANOVA). Significant differences between means were identified using Tukey’s post hoc test at *p* < 0.05. Statistical analyses were conducted in GraphPad Prism 9.0.0 (GraphPad Software, San Diego, CA, USA).

## Figures and Tables

**Table 1:** Inclusion and exclusion criteria in the systematic review.

Inclusion Criteria	Exclusion Criteria
Articles in English	Articles in other languages
Articles describing the antioxidant and antiglycemic properties of ranitidine, cimetidine, and famotidine	Articles not describing the antioxidant and antiglycemic properties of ranitidine, cimetidine, and famotidine
Research articles (in vitro, ex vivo, in vivo, and clinical studies), meta-analyses, and systematic literature reviews	Case studies and abstracts

## Data Availability

Data is contained within the article and supplementary material.

## Supplementary Materials

The following supporting information can be downloaded at: https://www.mdpi.com/article/10.3390/ph16091273/s1. Figure S1. Flowchart of the systematic review process (Prisma). Figure S2. Molecular docking analysis of ranitidine to BSA. Table S1. Anti-glycation properties of H2 receptor antagonists. Table S2. Binding affinities of the preferred docking poses of H2 receptor antagonists to BSA in a molecular docking simulation.
