# Supplementary material for: Antioxidant and Anti-Glycation Potential of H2 Receptor Antagonists—In Vitro Studies and a Systematic Literature Review
Source: Pharmaceuticals (Basel). 2023 Sep 8;16(9):1273. doi: 10.3390/ph16091273 (PMC10535796; doi:10.3390/ph16091273)
Supplement: Supplementary file 1 [file pharmaceuticals-16-01273-s001.zip › pharmaceuticals-2541343-supplementary.pdf]

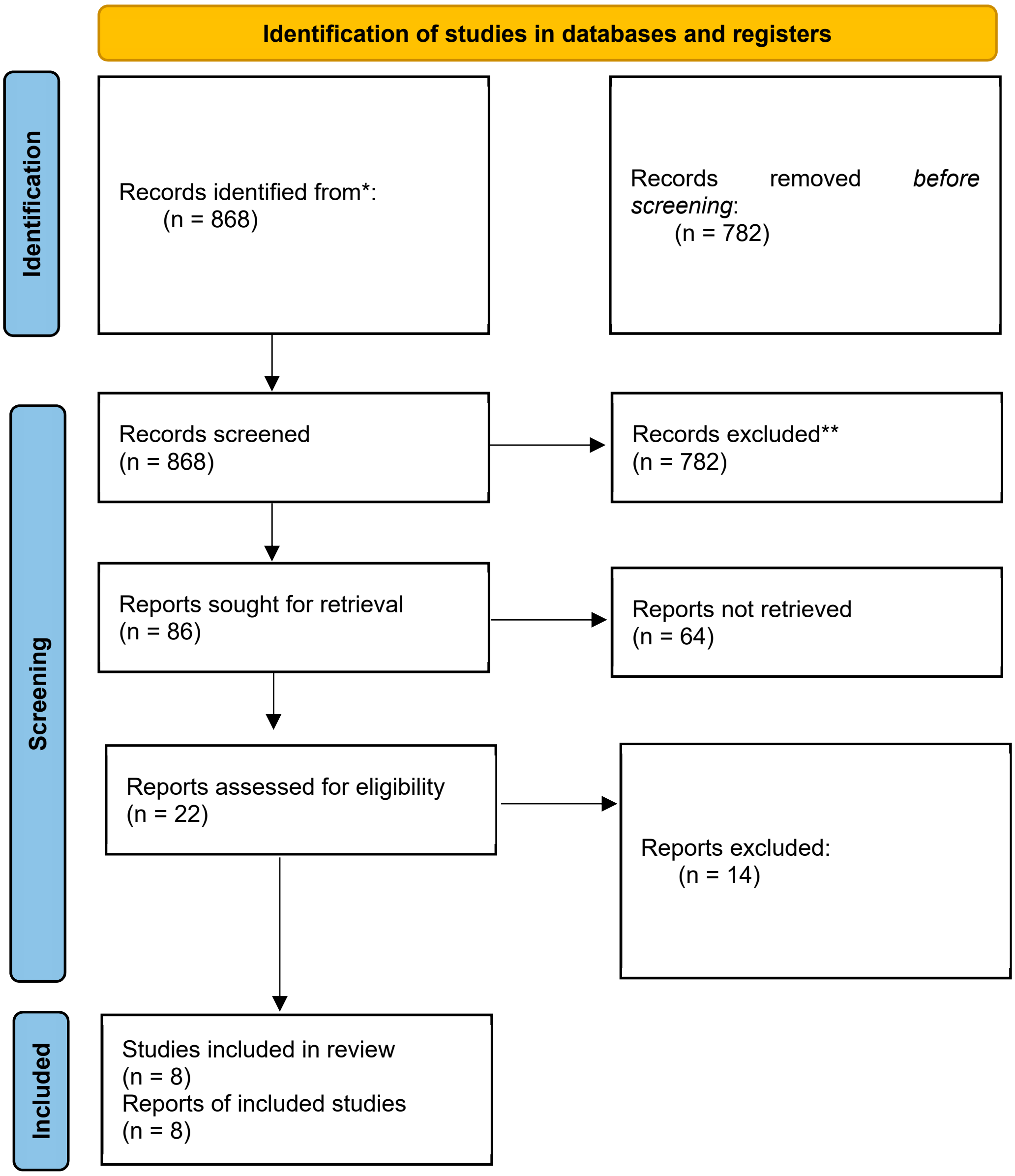

Figure S1. Flowchart of the systematic review process (Prisma).

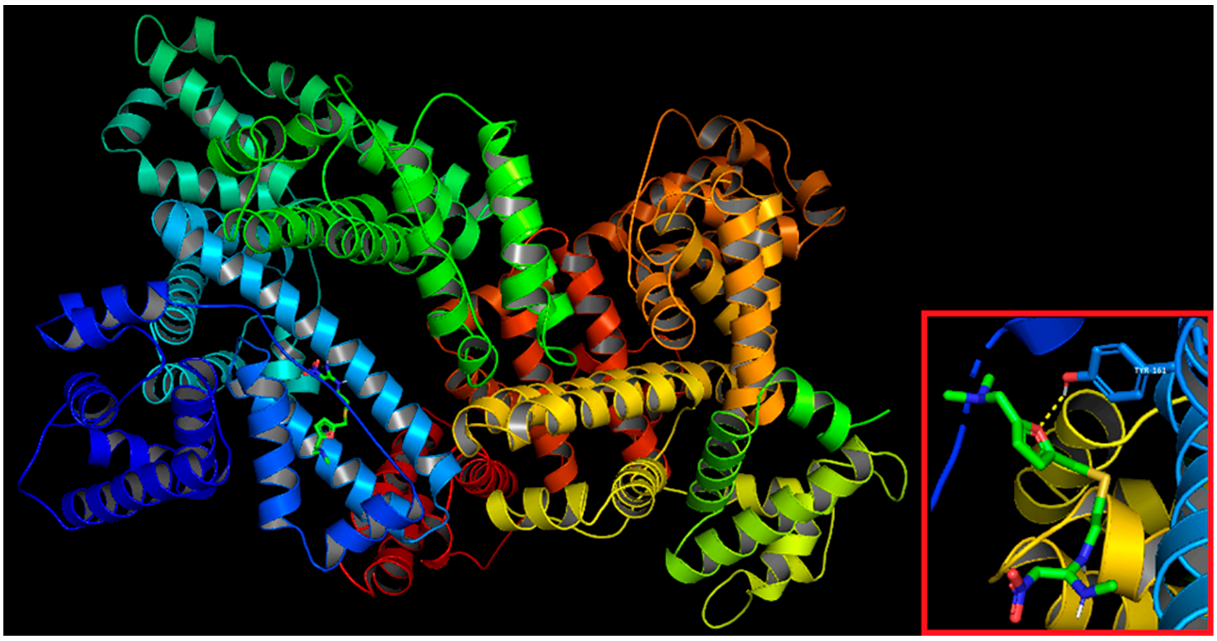

Figure S2. Molecular docking analysis of ranitidine to BSA.

Table S1. Anti-glycation properties of H2 receptor antagonists. ALT – alanine aminotransferase; AML – acute myeloid leukemia; AST – aspartate aminotransferase; CCl4 - carbon tetrachloride; DPPH - 1,1-diphenyl-2-picrylhydrazyl; fMLP - N-formyl-methionyl-leucyl-phenylalanine; H2O2 – hydrogen peroxide; HPLC - high-performance liquid chromatography; LPS – lipopolysaccharide; MDA – malondialdehyde; NADPH - nicotinamide adenine dinucleotide phosphate; NO – nitric oxide; ROS – reactive oxygen species; TBARS – thiobarbituric acid reactive substance; TNFα – tumor necrosis factor alpha.

Study design	Results	Endpoints	References
	In vitro/ex vivo		
Neutrophils isolated from 12 healthy volunteers were incubated with the following concentrations of H2 antagonists: cimetidine – 0, 1, 10, and 100 mg/mL; ranitidine – 0, 0.1, 1, and 10 mg/mL; and famotidine – 0, 0.02, 0.2, and 2 mg/mL.	The production of H_2O_2 was measured by quantifying the decrease in the fluorescence intensity of scopoletin which is oxidized in the presence of peroxidase. O_2^- generation by neutrophils was determined as the activity of reduced cytochrome C. Cimetidine and famotidine decreased the production of O_2^- and H_2O_2 in human neutrophils. Cimetidine decreased the rate of ROS generation at all concentrations, although fluorescence intensity decreased with a rise in cimetidine dose. ROS production was inhibited by famotidine doses of 0.2 and 2 mg/ml, and fluorescence intensity also decreased with a rise in dose.	Cimetidine and famotidine inhibit the production of superoxide (O_2^-) and H_2O_2 in human neutrophils.	[26]
Neutrophils stimulated by N-formyl-methionyl-leucyl-phenylalanine (fMLP) and suspended in PBS (cell density: 5 000 cells/ml) were exposed to ranitidine at a concentration of 10 and 100 mM	Ranitidine applied at a concentration of 100 mM considerably inhibited the release of neutrophil elastase from fMLP-stimulated neutrophils (the analytical method involved a chromogenic substrate). Ranitidine applied at a concentration of 10 mM inhibited superoxide (O_2^-) production by zymosan-induced opsonized neutrophils.	Ranitidine exerts indirect antioxidant effects by inhibiting neutrophil activation (decreased production of neutrophil elastase and O_2^-).	[27]
Monocytes suspended in the RPMI 1640 growth medium and 1% bovine calf serum were incubated for 2 hours at a temperature of 37°C in a humidified incubator (5% CO_2) and then stimulated with lipopolysaccharide (LPS) at 100 ng/ml for 4 h without or with H2 receptor antagonists applied at different concentrations. After incubation, cell suspensions were centrifuged at 10,000 g for 10 minutes. The concentration of the tumor necrosis factor alpha (TNFα) in the supernatant was determined in an immunoenzymatic assay.	Ranitidine applied at a concentration of 100 µM significantly inhibited TNFα production by LPS-stimulated monocytes. Famotidine did not inhibit TNFα production.	Ranitidine exerts indirect antioxidant effects by inhibiting TNFα production by LPS-stimulated monocytes.	[24]

An evaluation of cimetidine's $OH^{.}$ radical scavenging potential in gastric juice.	Cimetidine was a potent scavenger of hydroxyl radicals in the Fenton reaction with a rate constant of 14.8 × 10^{9} $M^{-1}s^{-1.}$	Cimetidine scavenges hydroxyl radicals ($OH^{.}$).	[28]
An evaluation of the $OH^{.}$ radical scavenging potential of ranitidine, cimetidine, and famotidine, applied at a final concentration 10, 28, 100 and 1000 µmol l^{-1}, in gastric juice.	In the deoxyribose test (which evaluates the reactivity of various compounds for the $OH^{.}$ radical based on the formation of $OH^{.}$ by the Fe^{2+}complex in the Fenton reaction), all drugs scavenged $OH^{.}$ with a high rate constant of 1·6 x 10^{10} $mol^{-1}s^{-1}$ for cimetidine, 7·5 x 10^{9} $mol^{-1}s^{-1}$ for ranitidine, and 1·7 x 10^{10} mol^{-1}s-^{1} for famotidine.	Ranitidine, cimetidine, and famotidine scavenge hydroxyl radicals ($OH^{.}$).	[29]
Gastric, liver, and brain homogenates of Wistar rats were exposed to famotidine concentrations of 0.1, 1.0 and 5.0 mM	Lipid peroxidation was evaluated by determining the concentration of thiobarbituric acid reactive substances (TBARS). Famotidine applied at a concentration of 1 mM stimulated lipid peroxidation (increase in TBARS value), but inhibited lipid peroxidation (decrease in TBARS value) when applied at a concentration of 5 mM.	Famotidine exerts varied effects on lipid peroxidation, depending on the applied dose (stimulatory effect at 1 mM, inhibitory effect at 5 mM).	[30]
Brain homogenates of male Wistar rats were exposed to cimetidine concentrations of 0.0, 0.5, 1.0, and 2.0 mM.	O_2^- generation was determined in a solution containing nitro blue tetrazolium (NBT) and cimetidine. Malondialdehyde (MDA) concentration was determined by high-performance liquid chromatography (HPLC). Increasing doses of cimetidine effectively reduced O_2^- generation (decrease in diformazan production) and inhibited lipid peroxidation (decrease in MDA concentration).	Cimetidine decreases O_2^- production and inhibits iron-dependent lipid peroxidation.	[31]
	In vivo		
A single dose of cimetidine (50, 100, 200 mg/kg BW), ranitidine (12.5, 25, 50, 100, 200 mg/kg BW), or famotidine (5, 10, 20, 40 mg/kg BW) was administered to male mice from the outbred stock of the Naval Medical Research Institute (NMRI)	In liver homogenates of rats administered cimetidine, ranitidine, and famotidine, the IC50 values for 1,1-diphenyl-2-picryl hydrazyl (DPPH) (concentration of the sample required to scavenge 50% of DPPH radicals) were determined at 671 ± 28, 538 ± 21, and 955 ± 43 µg / ml, respectively.	Ranitidine scavenged DPPH radicals more effectively than famotidine and cimetidine.	[22]
A single dose of cimetidine (50, 100, 200 mg/kg BW), ranitidine (12.5, 25, 50, 100, 200 mg/kg BW), or famotidine (5, 10, 20, 40 mg/kg BW) was administered to male mice from the outbred stock of the Naval Medical Research Institute (NMRI).	All drugs effectively captured nitric oxide (NO) in the range of 0.05 to 1.6 mg/ml. Their inhibitory effects increased with the applied dose. Famotidine was a highly potent scavenger (IC50 58 ± 3.2 µg/ml). Cimetidine was a weak scavenger (IC50 = 1.14 ±	H2 antagonists effectively scavenge NO radicals. Famotidine is a more potent scavenger of NO radicals than ranitidine and cimetidine.	[22]

	0.06 mg/ml). Ranitidine scavenged only 25% of NO radicals when administered at 1600 mg/ml.		
A single dose of cimetidine (50, 100, 200 mg/kg BW), ranitidine (12.5, 25, 50, 100, 200 mg/kg BW), or famotidine (5, 10, 20, 40 mg/kg BW) was administered to male mice from the outbred stock of the Naval Medical Research Institute (NMRI).	Cimetidine, ranitidine, and famotidine were not effective scavengers of hydrogen peroxide. Cimetidine was a more potent scavenger than ranitidine and famotidine, and its IC50 value was determined at 980 ± 35 µg / ml. Ranitidine was a very weak scavenger of hydrogen peroxide (IC50 = 1.25 ± 0.04 mg/ml), whereas famotidine did not exhibit any scavenging activity.	Cimetidine is a more potent scavenger of H_2O_2 than ranitidine and famotidine.	[22]
Rats with experimentally induced ischemia/reperfusion were intravenously administered ranitidine at 30 mg/kg BW and famotidine at 5 mg/kg BW 30 minutes before reperfusion. TNFα was measured in hepatocyte homogenates with the ELISA method.	Ranitidine administered intravenously at 30 mg/kg BW significantly decreased TNFα concentration at 1, 2, and 6 h after reperfusion. Famotidine administered at 5 mg/kg BW did not affect TNFα production.	Ranitidine exhibits indirect antioxidant activity by inhibiting TNFα production in the hepatocytes of rats with ischemia/reperfusion.	[24]
Male Wistar rats were intravenously administered ranitidine at 30 mg/kg BW. The rats were then placed in a restraint cage and immersed in water (22°C) up to the xiphoid process	TBARS concentration in the gastric mucosa was significantly lower in the ranitidine group than the control group (saline solution).	Ranitidine decreases lipid peroxidation in gastric mucosal injury induced by water immersion-restraint stress.	[27]

Table S2. Binding affinities of the preferred docking poses of H2 receptor antagonists to BSA in a molecular docking simulation.

	Mode	Affinity (kcal/mol)	RMSD (lower bond)	RMSD (upper bond)	Amino acid residues
**Ranitidine**	**1**	-6.0	0.000	0.000	TYR-161
	**2**	-5.6	29.470	31.414	ARG-117
	**3**	-5.3	30.985	32.438	HIS-146
	**4**	-5.2	30.169	32.308	HIS-146, TYR-161
	**5**	-5.2	28.629	30.848	LEU-115, ARG-186
	**6**	-5.2	30.724	32.219	LEU-115
	**7**	-5.2	31.502	32.750	---------
	**8**	-5.2	29.470	31.274	ARG-117, LEU-115
	**9**	-5.1	1.876	2.083	--------
**Cimetidine**	**1**	-5.8	0.000	0.000	TYR-161
	**2**	-5.7	1.490	2.354	ARG-186, TYR-161
	**3**	-5.6	3.013	4.612	LEU-182
	**4**	-5.5	3.882	5.201	--------
	**5**	-5.5	3.116	6.981	TYR-161
	**6**	-5.5	1.718	2.929	-------
	**7**	-5.5	29.271	31.205	TYR-161
	**8**	-5.5	2.615	8.250	PHE-134, TYR-161
	**9**	-5.5	38.572	40.393	VAL-293, LYS-444
**Famotidine**	**1**	-5.6	0.000	0.000	ASP-451, ASN-295, VAL-293
	**2**	-5.4	2.549	3.450	PRO-447
	**3**	-5.4	41.048	42.635	LEU-115, PHE-134, TYR-161
	**4**	-5.4	34.157	35.339	GLN-221
	**5**	-5.3	32.180	34.004	THR-515, ASP-183, SER-517
	**6**	-5.3	10.089	12.585	TYR-452, GLU-188
	**7**	-5.2	25.554	26.773	ASP-183, ARG-117, TYR-161
	**8**	-5.2	23.949	25.892	LEU-182, ASP-183
	**9**	-5.2	3.024	5.940	ALA-291, ARG-218, ASP-451, PRO-447
